# Effects of vitamin D supplementation on depression in adult women: a systematic review of randomized controlled trials

**DOI:** 10.7586/jkbns.25.092

**Published:** 2026-02-27

**Authors:** Byung-Jun Park, Sang-Dol Kim

**Affiliations:** Department of Nursing, College of Health Sciences, Kangwon National University, Samcheok, Korea

**Keywords:** Adult women, Depression, Systematic review, Vitamin D supplementation

## Abstract

**Purpose:**

This systematic review evaluated the effects of vitamin D supplementation on depressive symptoms in adult women by synthesizing evidence from randomized controlled trials (RCTs).

**Methods:**

This review was conducted in accordance with PRISMA 2020 guidelines. PubMed/MEDLINE, Web of Science, PsycINFO, and CINAHL were searched for RCTs published between January 2020 and November 2025. Eligible studies included women aged ≥ 18 years, vitamin D supplementation as the primary intervention, and assessment of depressive symptoms using validated instruments. Two independent reviewers performed study screening, data extraction, and quality assessment using the Cochrane risk of bias 2.0 tool.

**Results:**

Four RCTs (n = 18,628 participants) were included. Three trials enrolled women with type 2 diabetes or prediabetes and concomitant vitamin D deficiency, whereas one large trial enrolled adults aged ≥ 50 years without baseline depression. Interventions ranged from 2,000 IU daily to 50,000 IU weekly for 6 months to 5.3 years. Two trials demonstrated significant reductions in depressive symptoms with high-dose supplementation (25,000~50,000 IU weekly) in women with comorbidities and vitamin D deficiency (*p* < .050). One trial showed overall improvement, but no between-group differences. The large preventive trial (2,000 IU daily, 5.3 years) found no effect on depression incidence (hazard ratio, 0.97; 95% confidence interval, 0.87~1.09).

**Conclusion:**

High-dose vitamin D supplementation may be associated with reductions in depressive symptoms among women with vitamin D deficiency and metabolic comorbidities, whereas low-dose supplementation appears to confer limited preventive benefit in the general population.

## INTRODUCTION

Depression is among the most prevalent global mental health disorders, affecting more than 300 million individuals worldwide, with adult women experiencing nearly twice the prevalence observed in men [1]. It is a leading cause of disability and is associated with reduced quality of life, increased suicide risk, and substantial socioeconomic burden. Women remain at higher lifetime risk because of interacting biological, psychological, and social factors. Although antidepressant pharmacotherapy and psychotherapy serve as first-line treatment modalities, their efficacy is limited, with response rates of approximately 50.0%~60.0%. Adverse effects and relapse further underscore the need for complementary therapeutic strategies.

Growing interest has focused on nutritional influences on mental health, particularly vitamin D. Traditionally recognized for its role in calcium homeostasis and bone metabolism, vitamin D also exerts broader neurobiological effects [2]. Vitamin D receptors (VDRs) are expressed in several brain regions involved in mood regulation and neuroplasticity, including the hippocampus, prefrontal cortex, and hypothalamus. Additionally, vitamin D modulates serotonin synthesis through regulation of tryptophan hydroxylase expression, and has been associated with increased brain-derived neurotrophic factor (BDNF), which supports neuronal survival and function [2,3].

Epidemiological evidence consistently associates low vitamin D levels with depression. Meta-analyses have reported significant associations between vitamin D deficiency and increased risk of depressive symptoms [4], and prospective cohort studies have indicated that insufficient vitamin D levels predict subsequent depressive symptoms [5]. Adult women are particularly vulnerable to vitamin D deficiency because of hormonal changes associated with pregnancy, childbirth, and menopause, making them a priority population for supplementation research. Studies have reported high prevalence of vitamin D deficiency in women with metabolic conditions such as prediabetes and type 2 diabetes [6,7].

Despite this biological and epidemiological evidence, randomized controlled trials (RCTs) evaluating vitamin D supplementation for depression have produced inconsistent findings. Several trials have demonstrated improvements in depressive symptoms with vitamin D supplementation [6-8], whereas large long-term trials in general populations have found no significant preventive effect on depression incidence [9]. Previous systematic reviews have similarly reported inconclusive findings, and few have focused specifically on women or incorporated recently published trials. Updated synthesis is therefore needed to clarify the effects of vitamin D supplementation on depressive symptoms among adult women.

The decision to restrict the literature search to studies published between January 2020 and November 2025 was based on several methodological and clinical considerations. First, this period reflects current vitamin D supplementation practices, modern depression assessment tools, and contemporary clinical populations. Second, methodological advances in vitamin D research in the late 2010s, including standardization of serum 25-hydroxyvitamin D [25(OH)D] measurement and refinement of supplementation protocols, support emphasis on more recent studies. Third, the coronavirus disease 2019 pandemic substantially altered sun exposure patterns, dietary habits, and mental health prevalence, potentially limiting the applicability of pre-2020 findings. Fourth, heterogeneity in outcome measurement was reduced, as older studies frequently relied on outdated or non-standardized depression scales. Fifth, focusing on recent studies aligns with recommendations for rapid evidence synthesis and maintains clinical relevance. Finally, because pre-2020 evidence has already been comprehensively synthesized in previous systematic reviews and meta-analyses [4,8], this review was designed to update and extend the existing literature rather than duplicate prior work.

A systematic review of RCTs was selected to provide robust evidence on the effects of vitamin D supplementation on depressive outcomes. Randomization minimizes confounding and reduces the risk of reverse causation, such as depression leading to reduced outdoor activity and subsequently lower vitamin D levels. Systematic synthesis was necessary to reconcile inconsistent findings across individual trials. Conducting the review in accordance with Preferred Reporting Items for Systematic Reviews and Meta-Analyses (PRISMA) 2020 guidelines [10] ensured transparency, reproducibility, and methodological rigor, which are critical for informing clinical practice and health policy decisions. The purpose of this systematic review was to (1) evaluate the effects of vitamin D supplementation on depressive symptoms in adult women, (2) assess the methodological quality of the included studies, (3) examine moderating factors, including baseline vitamin D levels, supplementation dosage, intervention duration, and depression severity, and (4) provide implications for clinical practice and directions for future research.

## METHODS

### 1. Study design

This study was conducted in accordance with the PRISMA 2020 guidelines [10]. A research protocol was established prior to data collection. This systematic review protocol was not prospectively registered in PROSPERO (International Prospective Register of Systematic Reviews) or other public registry. The review was initiated as a time-sensitive project aimed at providing updated evidence on a rapidly evolving clinical question, and the additional time required for PROSPERO registration and approval would have delayed the project substantially. At the time of project initiation, protocol registration was encouraged but not universally required by all journals in the field. A detailed internal protocol was developed before data extraction and followed throughout the review process, and no substantial deviations from the planned methods occurred. Nevertheless, the absence of public protocol registration represents a limitation because it reduces transparency regarding potential protocol deviations. Future systematic reviews should prioritize prospective protocol registration in PROSPERO or similar registries to enhance transparency and reduce the risk of selective reporting or post-hoc methodological changes.

### 2. Search strategy

A comprehensive literature search was conducted in PubMed/MEDLINE, Web of Science, PsycINFO, and CINAHL databases to identify relevant studies published between January 1, 2020, and November 30, 2025. Search terms were developed using the Population, Intervention, Comparison, and Outcome (PICO) framework and included Medical Subject Headings (MeSH) and free-text keywords. The search strategy incorporated terms related to the population (“adult women,” “female”), intervention (“vitamin D,” “cholecalciferol,” “ergocalciferol,” “vitamin D supplementation”), outcome (“depression,” “depressive symptoms,” “major depressive disorder”), and study design (“randomized controlled trial,” “RCT,” “clinical trial”).

**Figure 1.**
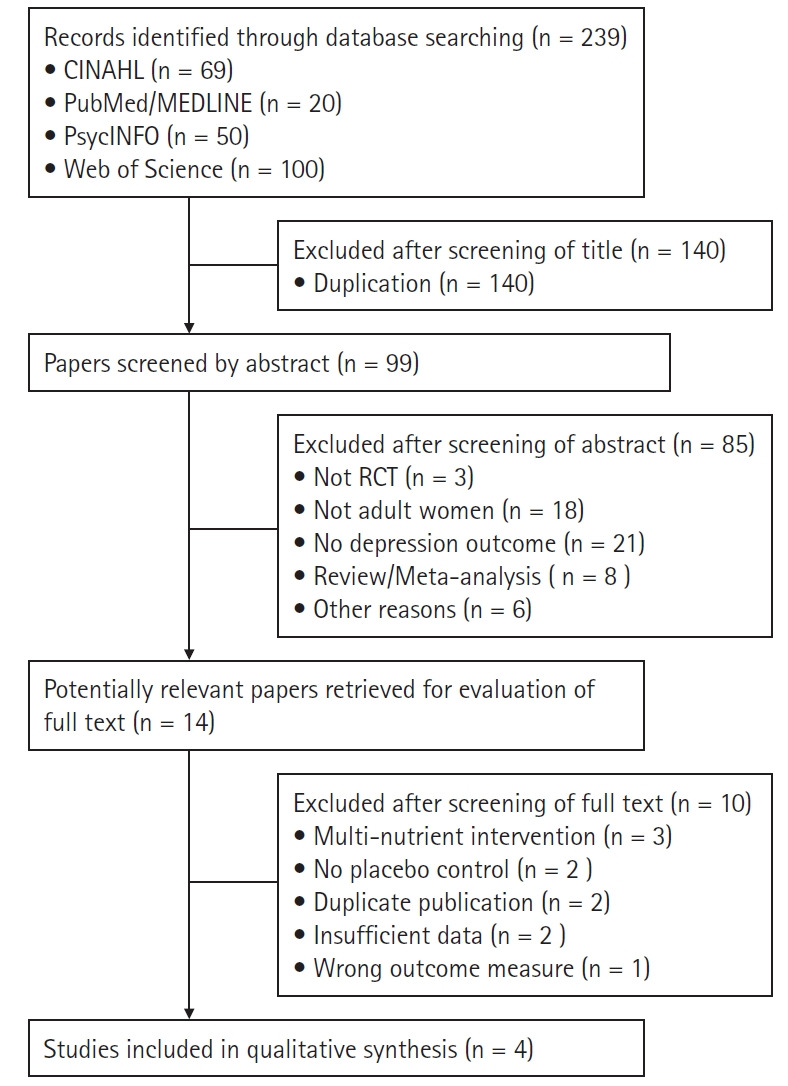
PRISMA 2020 flow diagram for study selection. PRISMA = Preferred Reporting Items for Systematic Reviews and Meta-Analyses; RCT = Randomized controlled trial.

The selected databases provided broad coverage of medical, psychological, and nursing literature relevant to the research question. The Cochrane Central Register of Controlled Trials (CENTRAL) was not searched because preliminary scoping indicated substantial overlap with the selected databases, particularly for recent vitamin D and depression trials, and resource constraints required prioritization. No formal hand searching or gray literature searches were conducted to focus on peer-reviewed, indexed studies. These decisions may have resulted in missed studies and are acknowledged as limitations. Search syntax was tailored to each database, and filters were applied to include RCTs within the specified timeframe. Title and abstract screening was performed independently by two reviewers, followed by full-text assessment for eligibility. Disagreements were resolved by consensus. Duplicate and irrelevant articles were removed. The study selection process was summarized using a PRISMA flow diagram (Figure 1).

### 3. Eligibility criteria

Studies were included if they met the following PICOS criteria: (1) Population: women aged ≥ 18 years; (2) Intervention: vitamin D supplementation (cholecalciferol or ergocalciferol) as the primary intervention; (3) Comparison: placebo or no-treatment control group; (4) Outcome: depressive symptoms assessed using validated instruments, such as Beck Depression Inventory (BDI), Patient Health Questionnaire (PHQ), Hamilton Depression Rating Scale (HDRS), or Center for Epidemiologic Studies Depression Scale (CES-D); and (5) Study design: RCTs published in English or Korean. Studies were excluded if they: (1) were review articles, meta-analyses, or systematic reviews; (2) were non-RCTs or observational studies; (3) included only male participants or pediatric populations; (4) involved multi-nutrient interventions without vitamin D as the primary intervention; (5) failed to report depression-related outcomes; or (6) were unpublished or non–peer-reviewed materials (e.g., conference abstracts or study protocols).

### 4. Data extraction

Two reviewers independently extracted data using a standardized form. Extracted information included: (1) study characteristics (first author, publication year, country); (2) study design and sample size; (3) participant demographics (age, baseline health status, baseline vitamin D levels, baseline depression severity); (4) intervention characteristics (vitamin D form, dosage, frequency, duration); (5) comparator type (placebo or no-treatment); (6) outcome measurement tools and time points; (7) depression outcomes (baseline and post-intervention scores, mean differences, standard deviations, effect sizes); (8) adverse events; (9) attrition rates; (10) trial registration; and (11) funding sources. When data were missing or unclear, authors were contacted for clarification.

### 5. Risk of bias assessment

The methodological quality of included studies was assessed using the Cochrane risk of bias 2.0 (RoB 2.0) tool [11], which evaluates five domains: (1) randomization process; (2) deviations from intended interventions; (3) missing outcome data; (4) measurement of the outcome; and (5) selection of the reported result. Each domain was evaluated as “low risk,” “some concerns,” or “high risk” of bias. Two reviewers independently performed the assessment, and disagreements were resolved through reviewer consensus. Overall risk of bias for each study was determined based on the highest risk level across all domains. Publication bias was examined when at least ten studies were available using funnel plot visualization and Egger’s regression test to detect asymmetry. However, due to the limited number of included studies, formal publication bias assessment could not be performed

### 6. Data analysis

Data analysis was planned using Review Manager (RevMan), version 5.4 (The Cochrane Collaboration, London, UK). For continuous outcomes, standardized mean differences with 95% confidence intervals (CIs) were calculated. Statistical heterogeneity was assessed using Cochran’s Q test and Higgins’ I^2^ statistic, with I^2^ values of 25%, 50%, and 75% indicating low, moderate, and high heterogeneity, respectively. A fixed-effects model was used for I^2^ < 50%, and a random-effects model for I^2^ ≥ 50%. Subgroup analyses were performed to explore potential sources of heterogeneity, including: (1) baseline vitamin D status (deficient [< 20 ng/mL] vs. sufficient [≥ 30 ng/mL]); (2) supplementation dosage (< 4,000 vs. ≥ 4,000 IU/day); (3) intervention duration (< 12 vs. ≥ 12 weeks); (4) age group (reproductive age vs. postmenopausal); and (5) baseline depression severity (mild vs. moderate to severe). Sensitivity analyses were conducted by removing studies with high risk of bias to assess the robustness of findings. However, due to substantial clinical and methodological heterogeneity among the included studies (variation in participant characteristics, supplementation regimens, and outcome measures), quantitative meta-analysis was not performed. Instead, a qualitative narrative synthesis was conducted, summarizing study characteristics, intervention effects, and factors influencing outcomes.

### 7. Ethical considerations

This study involved analysis of previously published, publicly available data and did not include direct interaction with human participants. Therefore, this study was exempt from formal Institutional Review Board (IRB) review. All included studies reported appropriate ethics approval and informed consent from participants in their original publications. No additional ethical concerns were identified during the conduct of this systematic review.

## RESULTS

### 1. Literature search and study selection

The database search identified 239 records from PubMed/MEDLINE (n = 20), Web of Science (n = 100), PsycINFO (n = 50), and CINAHL (n = 69). No additional records were identified from other sources. After removing 140 duplicates, 99 unique records remained for title and abstract screening. Of these, 85 records were excluded as they were not RCTs (n = 32), did not include adult women (n = 18), did not assess depression outcome (n = 21), were review or meta-analysis (n = 8), or for other reasons, including conference abstracts and study protocols (n = 6), leaving 14 articles for full-text eligibility assessment. Following full-text review, 10 articles were further excluded as they involved multi-nutrient interventions (n = 3), lacked a placebo control (n = 2), were duplicate publications (n = 2), provided insufficient data (n = 2), or used inappropriate outcome measures (n = 1). Ultimately, four RCTs met all inclusion criteria and were included in the qualitative synthesis [6,7,9,12]. The study selection process is shown in the PRISMA flow diagram (Figure 1, Appendix 1).

### 2. Characteristics of included studies

#### 1) Study design, publication year, and location

All included studies were RCTs published between 2020 and 2025. Three studies employed double-blind designs [7,9,12], and one used an open-label randomized controlled design [6]. The studies were conducted in Greece, Iran, and the United States. Two trials were conducted in Mediterranean and Middle Eastern regions [6,7], where vitamin D deficiency is highly prevalent. Zaromytidou et al. [6] reported vitamin D deficiency (< 20 ng/mL) in 92.68% of older adult women with prediabetes in Greece.

#### 2) Participants

Sample sizes ranged from 56 to 18,353 participants [7,9]. Participants were adult women or mixed-sex populations aged between 18 and 79 years. Three studies focused on women with specific health conditions: older women with prediabetes [6], adults with type 2 diabetes [7,12]. The large VITAL-DEP trial [9] enrolled adults aged ≥ 50 years (49% women) without baseline depression diagnosis. Baseline depression status varied: two trials enrolled women with mild-to-moderate depressive symptoms [6,7], one trial enrolled women with type 2 diabetes and depressive symptoms [12], and the VITAL-DEP trial evaluated participants without baseline depression diagnosis [9]. Baseline vitamin D levels also differed: three studies included participants with vitamin D deficiency (< 20 ng/mL) or insufficiency (20~30 ng/mL) [6,7,12], whereas the VITAL-DEP trial did not restrict enrollment by baseline vitamin D level (mean 30.7 ng/mL) [9].

#### 3) Intervention characteristics

All studies used vitamin D3 (cholecalciferol). Dosage regimens varied considerably: two trials used high-dose supplementation (25,000~50,000 IU weekly) for 8~12 months [6,7], one trial compared high-dose (50,000 IU weekly) versus low-dose (5,000 IU weekly) for 6 months [12], and the VITAL-DEP trial administered 2,000 IU daily for over 5 years [9]. All interventions were administered orally. Intervention duration ranged from 6 months to over 5 years.

### 3. Outcome measures

Depressive symptoms were measured using validated standardized instruments, including the Beck Depression Inventory-II (BDI-II), Patient Health Questionnaire-9 (PHQ-9), Patient Health Questionnaire-8 (PHQ-8), and Center for Epidemiologic Studies Depression Scale (CES-D). Depression symptom severity was assessed at baseline and post-intervention, with some studies also reporting interim assessments. All four trials reported post-intervention vitamin D levels (Table 1).

### 4. Effects of vitamin D supplementation on depressive symptoms

The effects of vitamin D supplementation on depressive symptoms varied substantially across studies, influenced by baseline vitamin D status, supplementation dosage and duration, and baseline depression severity.

#### 1) High-dose interventions in women with comorbid conditions

Two trials demonstrated significant reductions in depressive symptoms with high-dose vitamin D supplementation in women with vitamin D deficiency and comorbid metabolic conditions. Kaviani et al. [7] reported that vitamin D3 at 50,000 IU every two weeks for 8 weeks significantly reduced BDI-II scores in adults with type 2 diabetes and mild-to-moderate depression (mean change: −11.75 in the intervention group vs. −3.61 in the control group, p = .003). A weekly dose of 25,000 IU for 12 months significantly improved PHQ-9 scores in older adult women with prediabetes (12-month scores: 13.52 ± 5.01 in the intervention group vs. 20.20 ± 8.67 in the control group, *p* < .001) [6]. Two different vitamin D3 dosing regimens (50,000 IU weekly versus 5,000 IU weekly) were evaluated for 6 months in women with type 2 diabetes and depressive symptoms [12]. Both groups showed significant improvements in CES-D scores over time (mean decrease of 12.98 points, 95% CI −15.04 to −10.93, *p* < .001), but no significant difference was observed between the high-dose and low-dose groups. Baseline mean 25(OH)D level was 20.8 ng/mL, indicating vitamin D insufficiency (Table 1).

#### 2) Long-term low-dose interventions in general population

The large VITAL-DEP ancillary study enrolled 18,353 participants (49% women, mean age 67.7 years for women) without a baseline depression diagnosis and followed them for a median of 5.3 years [9]. Vitamin D3 at 2,000 IU daily did not significantly reduce the risk of depression or clinically relevant depressive symptoms compared with placebo (hazard ratio 0.97, 95% CI 0.87~1.09). No significant difference in PHQ-8 scores was observed between groups (difference 0.01, 95% CI 0.04~0.05). This trial did not restrict enrollment by baseline vitamin D status (mean baseline 25(OH)D 30.7 ± 10.0 ng/mL) or depression severity (Table 1).

### 5. Heterogeneity and effect modifiers

Substantial heterogeneity across studies precluded quantitative meta-analysis. Factors associated with treatment effects included.

#### 1) Baseline vitamin D status:

Baseline vitamin D status: Three trials enrolling participants with vitamin D deficiency (< 20 ng/mL) or insufficiency (20~30 ng/mL) [6,7,12] showed therapeutic effects, whereas the study without baseline restriction [9] did not demonstrate preventive effects.

#### 2) Supplementation dosage and duration

Trials using high-dose regimens (25,000~50,000 IU weekly) for 6~12 months [6,7,12] were conducted in women with vitamin D deficiency and comorbid conditions, with two showing significant between-group differences [6,7] and one showing overall improvement without between-group differences [12]. The long-term (> 5 years), low-dose regimen (2,000 IU daily) in the general population [9] was not associated with preventive effects.

#### 3) Baseline depression severity

Therapeutic effects were observed in trials enrolling participants with existing depressive symptoms at baseline (mild-to-moderate depression) [6,7,12], whereas the trial enrolling participants without baseline depression diagnosis [9] showed no preventive effect.

#### 4) Participant characteristics

The presence of comorbid conditions (type 2 diabetes, prediabetes) [6,7,12], age (mean age 50~73 years in three studies [6,9,12] versus 18~60 years in one study [7]), and baseline vitamin D status may have influenced treatment response. However, subgroup analyses were not feasible because of limited data and heterogeneity in study designs.

### 6. Risk of bias assessment

Using the Cochrane RoB 2.0 tool, three studies [6,7,9] were rated as having low risk of bias across all domains. One study [12] raised some concerns, primarily related to deviations from intended interventions (open-label design without placebo control) and missing outcome data. No study was judged to have high risk of bias. Overall, the included studies demonstrated good methodological quality. Detailed risk of bias assessments are presented in Table 2. A funnel plot could not be produced due to the limited number of included studies; however, the presence of a large neutral trial [9] reduces concern for publication bias.

## DISCUSSION

This systematic review synthesized evidence from four RCTs evaluating the effects of vitamin D supplementation on depressive symptoms in adult women. The findings reveal a nuanced relationship according to supplementation strategy and baseline characteristics. High-dose vitamin D supplementation (25,000~50,000 IU weekly for 6~12 months) improved depressive symptoms in women with vitamin D deficiency and comorbid metabolic conditions (type 2 diabetes or prediabetes) [6,7,12], although one trial found no significant difference between high-dose and low-dose vitamin D groups [12]. Conversely, long-term, low-dose supplementation (2,000 IU daily for > 5 years) did not reduce depression incidence or symptom severity in the general population without baseline vitamin D deficiency or depression diagnosis [9]. These findings suggest that the antidepressant effects of vitamin D supplementation depend on baseline vitamin D status, comorbid conditions, dosing regimen, and the presence of existing depressive symptoms.

### 1. Biological mechanisms

The observed findings are biologically plausible given the established role of vitamin D in central nervous system function. VDRs and vitamin D-activating enzyme 1α-hydroxylase are expressed in brain regions involved in mood regulation, including the hippocampus, prefrontal cortex, and hypothalamus [2]. Vitamin D influences neurotransmitter synthesis, particularly serotonin, through regulation of tryptophan hydroxylase, the rate-limiting enzyme in serotonin production [2]. Additionally, vitamin D is associated with increased BDNF levels, which supports neuroplasticity, neuronal survival, and synaptic function [3]. Furthermore, vitamin D exerts anti-inflammatory effects by reducing pro-inflammatory cytokines, such as interleukin-6 and tumor necrosis factor-α, which have been implicated in depression pathophysiology. The consistency between these mechanistic pathways and the clinical improvements observed in deficient populations supports a causal role for vitamin D in mood regulation.

### 2. Importance of baseline vitamin D status

Baseline vitamin D status emerged as a key determinant of treatment response. Three trials enrolling participants with vitamin D deficiency (< 20 ng/mL) or insufficiency (20~30 ng/mL) [6,7,12] consistently demonstrated improvements in depressive symptoms, whereas the study without baseline restriction [9] showed no effect. This pattern suggests that vitamin D supplementation is most effective as a corrective intervention for deficiency rather than as a universal preventive strategy. These findings align with the threshold hypothesis, which proposes that vitamin D–related neurobiological effects are optimized once serum levels reach sufficiency, with limited benefit beyond this threshold [13]. Identifying individuals with low baseline vitamin D levels may therefore be critical for maximizing clinical benefit and avoiding unnecessary supplementation.

### 3. Dose and duration optimization

Difference in dosing and duration of vitamin D supplementation for depression further explain the variability in outcomes across studies. High-dose regimens (25,000~50,000 IU weekly for 6~12 months) in women with vitamin D deficiency and comorbid conditions [6,7,12] were associated with therapeutic effects, with two trials demonstrating significant between-group differences [6,7]. In contrast, long-term, low-dose supplementation (2,000 IU daily) in the general population maintained vitamin D sufficiency but did not prevent depression onset or reduce symptom severity in individuals without baseline deficiency [9]. One trial comparing high-dose versus low-dose vitamin D found overall symptom improvement in both groups without significant between-group differences, suggesting that even lower doses (5,000 IU weekly) may be effective when baseline deficiency is corrected [12]. This distinction suggests that correction of vitamin D deficiency may be required to achieve therapeutic effects, whereas maintenance dosing alone may be insufficient to influence mood in individuals with adequate baseline status. However, the optimal dose and duration remain unclear and may vary depending on baseline vitamin D status, comorbid conditions, and individual patient characteristics. Future research should further evaluate dose–response relationships to refine optimal supplementation strategies.

### 4. Therapeutic versus preventive approach

The findings indicate that vitamin D supplementation may have therapeutic value for women with existing depressive symptoms and concurrent vitamin D deficiency but limited preventive benefit for the general population. The VITAL-DEP study [9], which enrolled participants without baseline depression diagnosis, found no reduction in depression incidence despite prolonged supplementation. In contrast, trials targeting individuals with baseline depressive symptoms reported improvements [6,7,12], suggesting that vitamin D may ameliorate existing symptoms but not prevent new-onset depression in individuals with adequate baseline vitamin D status. This distinction has important clinical implications, suggesting that vitamin D supplementation may be better positioned as an adjunctive treatment rather than a population-wide preventive intervention. Screening for vitamin D deficiency may therefore be most relevant in women presenting with depressive symptoms.

### 5. Considerations specific to women

Focusing on women is clinically relevant given sex-specific differences in depression risk and vitamin D metabolism. Hormonal fluctuations across the reproductive lifespan—including pregnancy, postpartum period, and menopause—influence both vitamin D metabolism and mood regulation. Pregnancy and lactation increase vitamin D requirements, and postpartum depression affects up to 15% of women [14]. Women are also more likely to experience vitamin D deficiency because of factors such as reduced sun exposure (e.g., cultural practices, indoor occupations), higher adiposity, and dietary restrictions. The included studies enrolled women with specific characteristics: older women with prediabetes [6], adults with type 2 diabetes [7,12], and a general population including middle-aged and older adults [9]. However, none of the included trials specifically examined vitamin D supplementation effects across different reproductive stages (pregnancy, postpartum, perimenopause, postmenopause). This highlights a substantial gap in the literature and underscores the need for women-specific trials across different life stages to inform tailored clinical recommendations.

### 6. Clinical implications

The findings of this review support several clinically relevant considerations for managing depression in adult women. Screening for vitamin D deficiency should be considered in women presenting with depressive symptoms, particularly those with risk factors such as chronic diseases (including type 2 diabetes and prediabetes), obesity, limited sun exposure, or dietary restrictions. Measurement of serum 25(OH)D levels can help identify women who may benefit from targeted supplementation.

For women with vitamin D deficiency (< 20 ng/mL) or insufficiency (20~30 ng/mL) and comorbid conditions such as diabetes or prediabetes, an individualized supplementation strategy is warranted. High-dose regimens (e.g., 25,000~50,000 IU weekly for 6~12 months) may be considered based on the evidence from included trials [6,7,12], followed by monitoring to achieve and maintain serum levels of at least 30 ng/mL. The evidence suggests that correction of vitamin D deficiency is important for symptom improvement, although the optimal dose may vary [12].

Vitamin D supplementation should be used as an adjunct to standard depression treatments rather than as a replacement. It may complement established interventions, including antidepressant medications and psychotherapy, particularly in women with documented vitamin D deficiency and comorbid metabolic conditions. Patients should be informed that vitamin D supplementation alone is not a treatment for depression and should not delay initiation of conventional therapies.

Current evidence does not support routine high-dose vitamin D supplementation for depression prevention in women without deficiency or baseline depressive symptoms. For the general population, maintaining adequate vitamin D status through a balanced diet, appropriate sun exposure, and standard daily supplementation (600~800 IU) is a reasonable approach and avoids unnecessary exposure to high doses.

Safety monitoring is recommended when high-dose vitamin D supplementation is prescribed. Serum calcium levels and renal function should be monitored during high-dose therapy to detect adverse effects. Long-term vitamin D intake exceeding 10,000 IU per day should be avoided to minimize toxicity risk, and patients should be advised against unsupervised dose escalation.

### 7. Limitations

Several limitations should be considered when interpreting the findings of this review. Substantial heterogeneity across the included studies limited direct comparability and precluded quantitative meta-analysis. Differences in participant characteristics, including age ranges, baseline health status, and comorbidities, as well as supplementation regimens (dosage, frequency, and duration) and depression assessment tools, reduced the precision of effect estimates and generalizability of findings.

The small number of eligible studies (n = 4) represents a significant limitation. Three of the four included studies enrolled participants with specific comorbid conditions (type 2 diabetes or prediabetes) [6,7,12], which may limit generalizability to healthy women or those with other health conditions. The VITAL-DEP trial included both men and women (49% women) [9], and sex-specific analyses were not reported. The small number of eligible studies prevented formal assessment of publication bias using funnel plots or Egger’s regression. Although the inclusion of a large neutral trial [9] reduces concern for selective publication, the possibility that smaller null studies remain unpublished cannot be excluded.

Most included trials were relatively short in duration, with follow-up ranging from 6 months to 12 months [6,7,12], except for one large long-term study with median follow-up of 5.3 years [9]. Consequently, the persistence of beneficial effects following discontinuation of supplementation remains unclear, as does the optimal duration of maintenance therapy for women who initially respond to treatment. Questions about whether vitamin D supplementation provides lasting benefits or requires continuous administration to maintain symptom improvement cannot be adequately addressed with the current evidence base.

Mechanistic data were limited, as most trials focused on clinical outcomes such as depression symptom scores without consistently measuring biological markers such as BDNF, inflammatory cytokines, neurotransmitter activity, or neuroimaging correlates. This constrains understanding of the pathways through which vitamin D supplementation may influence depressive symptoms.

Our search strategy, while comprehensive, did not include all possible databases. Embase and the Cochrane Central Register of Controlled Trials were not searched, and no formal hand searching or gray literature review was conducted. These decisions may have resulted in missed studies, particularly those published in regional or non-indexed sources. Restricting the search to studies published between 2020 and 2025 may have excluded earlier relevant trials. However, this approach prioritized contemporary methodologies and populations and built upon prior reviews that synthesized pre-2020 evidence. Finally, the absence of prospective PROSPERO registration represents a limitation in transparency. Although a detailed internal protocol was established and followed without substantial deviation, public registration would have allowed independent verification of prespecified methods.

### 8. Future research directions

Future research should prioritize large, adequately powered RCTs designed specifically for women. Stratification by baseline vitamin D status (deficient, insufficient, and sufficient) and depression severity (mild, moderate, and severe) would allow identification of subgroups most likely to benefit from supplementation. Dose–response studies directly comparing supplementation regimens are needed to establish optimal dosing strategies. Head-to-head comparisons of daily versus intermittent dosing and varying dose levels should assess efficacy, safety, and adherence. Additionally, trials should include diverse populations, including healthy women without comorbid conditions, to enhance generalizability.

Life-course–focused research is warranted to examine vitamin D supplementation effects across reproductive stages, including pregnancy, postpartum period, perimenopause, and postmenopause, given stage-specific physiological and hormonal influences. Mechanistic studies incorporating biological markers, such as BDNF, inflammatory markers (e.g., interleukin-6, tumor necrosis factor-alpha, and C-reactive protein), neurotransmitter levels (e.g., serotonin and dopamine metabolites), and neuroimaging techniques would help clarify pathways through which vitamin D exerts its effects on mental health.

Standardization of depression assessment methodologies across studies would enhance the comparability and interpretability of findings. Future research should employ consistent, validated depression measurement tools and establish standardized time points for outcome assessment (e.g., baseline, 4 weeks, 8 weeks, 12 weeks, and 6 months). Additionally, studies should report both categorical outcomes (e.g., response and remission rates) and continuous measures (symptom severity scores) to provide a comprehensive picture of treatment effects [15,16]. Harmonization of outcome measures would facilitate future meta-analyses and enable more robust evidence synthesis.

Long-term follow-up studies are needed to evaluate the persistence of beneficial effects following discontinuation of supplementation, optimal duration of maintenance therapy, and long-term safety of various supplementation regimens. These studies should extend beyond the typical 6-month to 12-month timeframes to assess whether vitamin D supplementation provides lasting benefits or requires continuous administration. Additionally, long-term safety monitoring should include assessment of potential adverse effects, such as hypercalcemia, kidney stones, and cardiovascular events, particularly with higher supplementation doses.

## CONCLUSION

This systematic review of four RCTs indicates that high-dose vitamin D supplementation (25,000~50,000 IU weekly for 6~12 months) may improve depressive symptoms in adult women with vitamin D deficiency and comorbid metabolic conditions (type 2 diabetes or prediabetes), whereas long-term, low-dose supplementation (2,000 IU daily) shows limited preventive benefit in the general population without baseline vitamin D deficiency or depression. Treatment response appears to depend on baseline vitamin D status, comorbid conditions, supplementation regimen, and the presence of depressive symptoms at baseline. Screening for vitamin D deficiency may be considered in women presenting with depression, particularly those with metabolic comorbidities, and supplementation should be used as an adjunct to standard evidence-based treatments rather than as a substitute. Future studies should prioritize large-scale, women-specific RCTs across diverse populations and health conditions, stratified by baseline vitamin D status and depression severity, to refine dosing strategies and identify populations most likely to benefit from supplementation.

## Figures and Tables

**Table 1.** Characteristics of the Included Studies (N=4)

Study	Author (year)	Country	Sample size (I/C)	Population	Age (years)	Baseline vitamin D (ng/mL)	Intervention	Duration	Depression measure	Main results
A1	Kaviani et al. (2022) [7]	Iran	28/28	Adults with T2DM and mild-moderate depression	18~60	Mean baseline 25(OH)D measured; participants had low vitamin D	Vitamin D3 50,000 IU every 2 weeks vs. placebo	8 weeks	BDI-II	Significant reduction in BDI-II: −11.75 ± 6.40 (intervention) vs. −3.61 ± 10.40 (control), *p* = .003
A2	Zaromytidou et al. (2022) [6]	Greece	45/45	Elderly women with prediabetes and vitamin D deficiency	Mean 73 ± 7	< 20 ng/mL in 92.68% of participants	Vitamin D3 25,000 IU weekly vs. no treatment	12 months	PHQ-9	Significant reduction in PHQ-9 scores at 12 months: 13.52 ± 5.01 (intervention) vs. 20.20 ± 8.67 (control), *p* < .001
A3	Penckofer et al. (2022) [12]	United States	64/65	Women with T2DM and depressive symptoms	Mean 58.4 ± 9.3	Mean 20.8 ± 8.1 (insufficient)	Vitamin D3 50,000 IU weekly vs. 5,000 IU weekly (active control)	6 months	CES-D	Overall CES-D scores improved in both groups (mean decrease 12.98 points, 95% CI −15.04 to −10.93, *p* < .001); no significant difference between high-dose and low-dose groups
A4	Okereke et al. (2020) [9]	United States	9,011/9,342	General population (49% women), no baseline depression diagnosis	Mean 67.7 ± 7.1 (women)	Mean 30.7 ± 10.0; no restriction by baseline vitamin D status	Vitamin D3 2,000 IU daily vs. placebo	Median 5.3 years	PHQ-8	No significant effect on depression risk: HR 0.97 (95% CI 0.87~1.09); no difference in PHQ-8 scores: 0.01 (95% CI −0.04 to 0.05)

I = Intervention group; C = Control group; T2DM = Type 2 diabetes mellitus; IU = International units; BDI-II = Beck Depression Inventory-II; PHQ-9 = Patient Health Questionnaire-9; PHQ-8 = Patient Health Questionnaire-8; CES-D = Center for Epidemiologic Studies Depression Scale; HR = Hazard ratio; CI = Confidence interval; 25(OH)D = 25-hydroxyvitamin D.

**Table 2.** Risk of Bias Assessment Using Cochrane RoB 2.0 Tool

Study	Author (year)	Randomization process	Deviations from intended interventions	Missing outcome data	Measurement of outcome	Selection of reported result	Overall risk
A1	Kaviani et al. (2022) [7]	Low	Low	Low	Low	Low	Low
A2	Zaromytidou et al. (2022) [6]	Low	Low	Low	Low	Low	Low
A3	Penckofer et al. (2022) [12]	Low	Some concerns (open-label design without placebo control)	Some concerns (higher attrition in one group)	Low	Low	Some concerns
A4	Okereke et al. (2020) [9]	Low	Low	Low	Low	Low	Low

RoB 2.0 = Risk of Bias 2.0 tool; Low = Low risk of bias; Some concerns = Some concerns about risk of bias; High = High risk of bias (not applicable in this review).

## Data Availability

The datasets generated and/or analyzed during the current study are available from the corresponding author on reasonable request.

## Appendix 1. Studies included in the systematic review

A1. Kaviani M, Nikooyeh B, Etesam F, Jahangiri Behnagh S, Mohammadi Kangarani H, Arefi M, et al. Effects of vitamin D supplementation on depression and some selected pro-inflammatory biomarkers: a double-blind randomized clinical trial. BMC Psychiatry. 2022;22(1):694. https://doi.org/10.1186/s12888-022-04305-3

A2. Zaromytidou E, Koufakis T, Dimakopoulos G, Drivakou D, Konstantinidou S, Rakitzi P, et al. Vitamin D alleviates anxiety and depression in elderly people with prediabetes: a randomized controlled study. Metabolites. 2022;12(10):884. https://doi.org/10.3390/metabo12100884

A3. Penckofer S, Ridosh MM, Adams WG, Grzesiak M, Woo JG. Vitamin D supplementation for the treatment of depressive symptoms in women with type 2 diabetes: a randomized clinical trial. Journal of Diabetes Research. 2022;2022:4090807. https://doi.org/10.1155/2022/4090807

A4. Okereke OI, Reynolds CF, Mischoulon D, Chang G, Vyas CM, Cook NR, et al. Effect of long-term vitamin D_3_ supplementation vs placebo on risk of depression or clinically relevant depressive symptoms and on change in mood scores: a randomized clinical trial. JAMA. 2020;324(5):471-480. https://doi.org/10.1001/jama.2020.10224
